# Prognostic values of the integrated model incorporating the volume of metastatic regional cervical lymph node and pretreatment serum Epstein–Barr virus DNA copy number in predicting distant metastasis in patients with N1 nasopharyngeal carcinoma

**DOI:** 10.1186/s40880-017-0264-x

**Published:** 2017-12-29

**Authors:** Ji-Jin Yao, Guan-Qun Zhou, Ya-Qin Wang, Si-Yang Wang, Wang-Jian Zhang, Ya-Nan Jin, Fan Zhang, Li Li, Li-Zhi Liu, Zhi-Bin Cheng, Jun Ma, Zhen-Yu Qi, Ying Sun

**Affiliations:** 1 Department of Radiation Oncology, State Key Laboratory of Oncology in South China, Collaborative Innovation Center for Cancer Medicine, Sun Yat-sen University Cancer Center, Guangzhou, Guangdong 510060 P. R. China; 2 Department of Radiation Oncology, The Fifth Affiliated Hospital of Sun Yat-sen University, Zhuhai, Guangdong 519001 P. R. China; 3 Department of Medical Statistics and Epidemiology & Health Information Research Center & Guangdong Key Laboratory of Medicine, School of Public Health, Sun Yat-sen University, Guangzhou, Guangdong 510080 P. R. China; 4 Department of Imaging Diagnosis and Interventional Center, State Key Laboratory of Oncology in South China, Collaborative Innovation Center for Cancer Medicine, Sun Yat-sen University Cancer Center, Guangzhou, Guangdong 510060 P. R. China

**Keywords:** Nasopharyngeal carcinoma, Lymph node volume, Epstein–Barr virus DNA, Distant metastasis, Prognostic model

## Abstract

**Background:**

According to the 7th edition of the American Joint Committee on Cancer (AJCC) staging system, over 50% of patients with nasopharyngeal carcinoma (NPC) have N1 disease at initial diagnosis. However, patients with N1 NPC are relatively under-researched, and the metastasis risk of this group is not well-stratified. This study aimed to evaluate the prognostic values of gross tumor volume of metastatic regional lymph node (GTVnd) and pretreatment serum copy number of Epstein–Barr virus (EBV) DNA in predicting distant metastasis of patients with N1 NPC, and to develop an integrated prognostic model that incorporates GTVnd and EBV DNA copy number for this group of patients.

**Methods:**

The medical records of 787 newly diagnosed patients with nonmetastatic, histologically proven N1 NPC who were treated at Sun Yat-sen University Cancer Center between November 2009 and February 2012 were analyzed. Computed tomography-derived GTVnd was measured using the summation-of-area technique. Blood samples were collected before treatment to quantify plasma EBV DNA. The receiver operating characteristic (ROC) curve analysis was used to evaluate the cut-off point for GTVnd, and the area under the ROC curve was used to assess the predicted validity of GTVnd. The survival rates were assessed by Kaplan–Meier analysis, and the survival curves were compared using a log-rank test. Multivariate analysis was conducted using the Cox proportional hazard regression model.

**Results:**

The 5-year distant metastasis-free survival (DMFS) rates for patients with GTVnd > 18.9 vs. ≤ 18.9 mL were 82.2% vs. 93.2% (*P* < 0.001), and for patients with EBV DNA copy number > 4000 vs. ≤ 4000 copies/mL were 83.5% vs. 93.9% (*P* < 0.001). After adjusting for GTVnd, EBV DNA copy number, and T category in the Cox regression model, both GTVnd > 18.9 mL and EBV DNA copy number > 4000 copies/mL were significantly associated with poor prognosis (both *P* < 0.05). According to combination of GTVnd and EBV DNA copy number, all patients were divided into low-, moderate-, and high-risk groups, with the 5-year DMFS rates of 96.1, 87.4, and 73.8%, respectively (*P* < 0.001). Multivariate analysis confirmed the prognostic value of this model for distant metastatic risk stratification (hazard ratio [HR], 4.17; 95% confidence interval [CI] 2.34–7.59; *P* < 0.001).

**Conclusions:**

GTVnd and serum EBV DNA copy number are independent prognostic factors for predicting distant metastasis in NPC patients with N1 disease. The prognostic model incorporating GTVnd and EBV DNA copy number may improve metastatic risk stratification for this group of patients.

## Introduction

The highest incidence of nasopharyngeal carcinoma (NPC) occurs in South China, with an annual incidence of 15–50 cases per 100,000 population [1]. Due to anatomic constraints and high radiosensitivity of NPC, radiotherapy is the mainstay treatment modality for all patients with locoregional NPC. The introduction of intensity-modulated radiation therapy (IMRT) was a pioneering breakthrough that significantly improved local control of NPC [2, 3]. Currently, the locoregional control rate of NPC treated with IMRT is greater than 90% [4]; distant metastasis is now the main failure pattern [5, 6].

The N (node) category of the tumor-node-metastasis (TNM) staging system is the most reliable tool for assessing distant metastasis risk of NPC [7]. Since the negative cervical lymph nodes with retropharyngeal lymph node (RLN) metastasis that was classified as N0 disease in the 6th edition of the American Joint Committee on Cancer (AJCC) staging system was upgraded to N1 disease in the 7th edition [8], the proportion of N1 disease was projected to rise. Moreover, several studies have found that over 50% of patients with NPC presented with N1 disease at initial diagnosis based on the 7th edition of the AJCC staging system [9, 10]. However, patients with N1 NPC are relatively under-researched, and the metastasis risk of this group is not well-stratified.

A recent study by Lu et al. [11] showed that the gross tumor volume of the lymph nodes (GTVnd) was a significant factor affecting distant metastasis in NPC patients. Additionally, previous studies have demonstrated that pretreatment serum Epstein–Barr virus (EBV) DNA copy number is also a reliable predictor for metastasis of NPC [12, 13]. However, no prognostic model for the prognostic prediction has been investigated to date in patients with N1 NPC. In the present study, we therefore aimed to develop an integrated prognostic model that incorporates GTVnd and serum EBV DNA copy number to stratify metastasis risk of patients with N1 NPC and evaluate the value of this prognostic model.

## Patients and methods

### Patients

All patients included in the present study were treated at Sun Yat-sen University Cancer Center between November 2009 and February 2012. Inclusion criteria were as follows: being pathologically diagnosed with non-keratinizing or undifferentiated carcinoma of the nasopharynx; having N1 disease; without evidence of distant metastasis; receiving radical IMRT at initial diagnosis; and with available data of GTVnd and pretreatment serum EBV DNA copy number. This retrospective study was conducted in compliance with the institutional policy to protect the patients’ private information and was approved by the Institutional Review Board of Sun Yat-sen University Cancer Center. The authenticity of this article has been validated by uploading the key raw data onto the Research Data Deposit (RDD) public platform (http://www.researchdata.org.cn), with the approval RDD Number as RDDA2017000302.

All patients underwent a pretreatment evaluation which included a complete physical examination, magnetic resonance imaging (MRI) of the nasopharynx and neck, chest radiography, abdominal sonography, electrocardiography, bone scan, and complete blood sampling to examine cell counts, biochemical profile, and serum EBV DNA copy number measurement. Diseases were restaged by two radiation oncologists specializing in head and neck cancer according to the 7th edition of AJCC staging system [8], with disagreements resolved by consensus.

### EBV DNA measurement

As described in previous studies [14–16], serum EBV DNA copy number was measured by quantitative polymerase chain reaction (q-PCR) before treatment. A cut-off copy number of 4000 copies/mL was chosen to define low and high EBV DNA copy number, as this threshold has been shown to be prognostic in previous NPC studies using the same measurement system [17–19].

### The measurement of GTVnd

The imaging protocol of MRI was the same as that previously described [10]. MR images were reviewed independently by two radiologists with more than 10 years of experience; disagreements were resolved by consensus. The diagnostic criteria of GTVnd was as follows: (1) any cervical lymph node with a minimal axial diameter ≥ 10 mm (level Ib and IIa, ≥ 11 mm); (2) lymph nodes of any size with central necrosis or a contrast-enhanced rim; and (3) lymph nodes of any size with extracapsular spreading. The GTVnd was manually outlined on the planning system by a radiation oncologist and was then verified by another radiation oncologist who was specialized in NPC treatment. The involved retropharyngeal lymph nodes (RLNs) were included as part of the gross tumor volume of the primary tumor (GTVp), as a clear distinction between the RLNs and primary tumor remains difficult in NPC [20–22]. The GTVnd were calculated using the planning system with the summation-of-area technique, which multiplies the entire areas by the image reconstruction interval of 3 mm.

### Treatment and follow-up

All patients were treated according to the principle of treatment for NPC at Sun Yat-sen University Cancer Center. The target delineation and prescribed doses of radiotherapy and chemotherapeutic regimens were the same as that described previously [23].

Patients were followed up every 3 months during the first 3 years, every 6 months during the next 2 years, and annually thereafter. Routine follow-up included complete head and neck examination, nasopharyngoscopy, hematology and biochemical profiles, chest radiography, and abdominal sonography. Bone scan and computed tomography (CT) of the chest or abdomen and even positron emission tomography (PET)/CT were performed when clinically indicated, especially for patients with suspected distant metastasis. DMFS was defined as the period from the first treatment to the first report of distant metastasis or to the last follow-up. The patients who were lost to follow-up or alive without any events at the last follow-up were censored. Distant metastasis was confirmed by pathologic biopsy or no less than two imaging methods in favor of distant metastasis.

### Statistical methods

Receiver operating characteristic (ROC) curve was used to identify the cut-off point and test the prognostic validity of the GTVnd. The differences of patient characteristics between low and high EBV DNA copy number groups were compared using Pearson’s Chi square test. Cumulative survival rates were calculated by using the Kaplan–Meier method. A log-rank test was used to test the difference between cumulative survival rates with respect to risk groups classified according to clinical variables. A Cox proportional hazards regression model was applied to test the independent significance of different factors. Two-tailed *P* values < 0.05 were considered statistically significant. All analyses were performed using the R software version 3.1.2 (Vienna, Austria; https://mirrors.tuna.tsinghua.edu.cn/CRAN/).

## Results

### Patient characteristics

The clinical data of 1062 NPC patients who met all criteria were analyzed. Of the 1062 patients, 275 patients were excluded from the study, including 216 patients whose information of baseline EBV DNA copy number was incomplete and 59 patients whose GTVnd data were not available. The study group therefore contained 787 patients. For all these patients, the median GTVnd was 10.7 mL (interquartile range [IQR] 5.7–14.9 mL) for T1 NPC, 12.3 mL (IQR 6.7–17.9 mL) for T2 NPC, 12.4 mL (IQR 4.5–19.5 mL) for T3 NPC, and 16.8 mL (IQR 5.1–21.4 mL) for T4 NPC. The optimal cut-off value of GTVnd for DMFS prediction was 18.9 mL, with sensitivity of 0.83, specificity of 0.69, and area under the ROC curve of 0.73. Patients were divided into two groups according to the cut-off of GTVnd: small GTVnd group (GTVnd ≤ 18.9 mL) and large GTVnd group (GTVnd > 18.9 mL). Patients with an EBV DNA copy number of > 4000 copies/mL more commonly had large GTVnd, more frequently had advanced T-category (T3–4) diseases, and more often received neoadjuvant chemotherapy plus concurrent chemoradiotherapy (*P* < 0.001 for all; Table 1).

**Table 1:** Associations between serum EBV DNA copy number and GTVnd as well as clinical demographic characteristics of patients with N1 nasopharyngeal carcinoma

Characteristic	EBV DNA copy number^a^		*P* value
	≤ 4000 copies/mL (*n* = 477)	> 4000 copies/mL (*n* = 310)	
GTVnd (mL)			< 0.001
≤ 18.9	415 (87.0)	223 (71.9)	
> 18.9	62 (13.0)	87 (28.1)	
Gender			0.142
Male	336 (70.4)	234 (75.5)	
Female	141 (29.6)	76 (24.5)	
Age (years)			0.558
> 45	215 (45.1)	147 (47.4)	
≤ 45	262 (54.9)	163 (52.6)	
T category			< 0.001
T1–2	164 (34.4)	57 (18.4)	
T3–4	313 (65.6)	253 (81.6)	
Treatment method			< 0.001
Radiotherapy alone	55 (11.5)	14 (4.5)	
CCRT	280 (58.7)	130 (41.9)	
NACT + CCRT	142 (29.8)	166 (53.5)	

*EBV* pretreatment Epstein–Barr virus, *GTVnd* gross tumor volume of lymph nodes, *CCRT* concurrent chemoradiotherapy, *NACT* neoadjuvant chemotherapy

^a^All values are presented as number of patients followed by percentage in parentheses

### The outcomes of DMFS

**Fig. 1:**
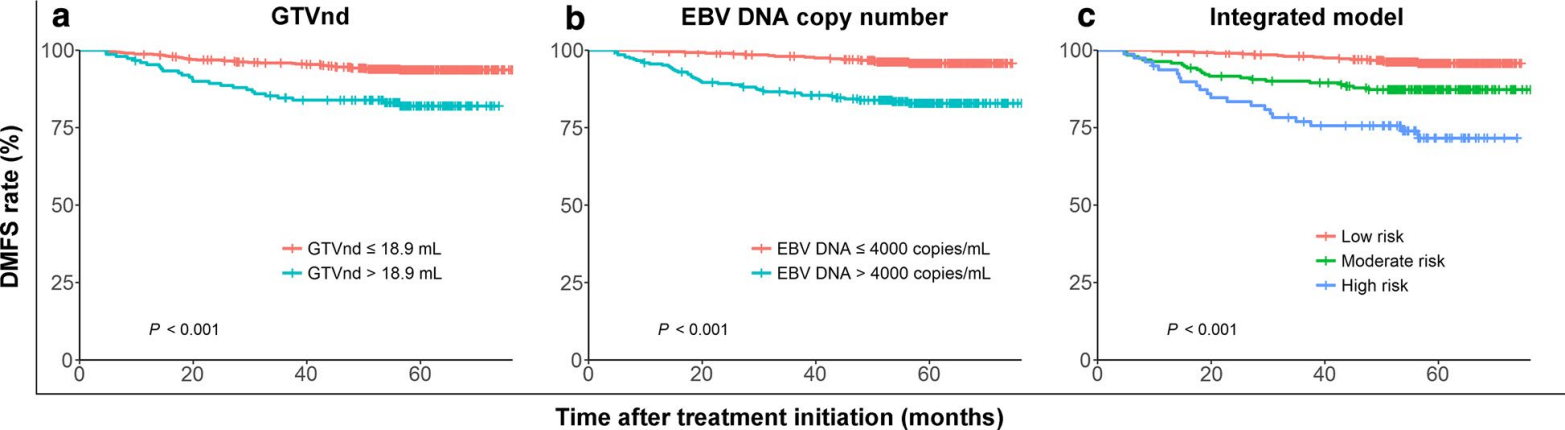
Kaplan–Meier distant metastasis-free survival curves for patients with N1 nasopharyngeal carcinoma stratified by **a** gross tumor volume of lymph node (GTVnd), **b** serum Epstein–Barr virus (EBV) DNA copy number, and **c** prognostic model. Low risk: EBV DNA ≤ 4000 copies/mL regardless of GTVnd; moderate risk: GTVnd ≤ 18.9 mL with EBV DNA > 4000 copies/mL; high risk: GTVnd > 18.9 mL with EBV DNA > 4000 copies/mL. *DMFS* distant metastasis-free survival

The median follow-up time was 59.2 months (range: 4.5–76.3 months). Distant metastasis was observed in 68 (8.6%) patients: 26 in the bone, 17 in the lung, 15 in the liver, and 10 in multiple sites. The 5-year DMFS rate for all patients was 91.4% (95% confidence interval [CI] 84.7%–96.2%). The DMFS rates for patients stratified by GTVnd and EBV DNA copy number are shown in Fig. 1. Among patients with N1 disease, the 5-year DMFS rate was lower in patients with GTVnd > 18.9 mL than in patients with GTVnd ≤ 18.9 mL (82.2% vs. 93.2%, *P* < 0.001; Fig. 1a). In addition, patients with an EBV DNA copy number of > 4000 copies/mL had an increased risk of distant metastasis compared with those with an EBV DNA copy number of equal to or less than 4000 copies/mL (83.5% vs. 93.9%, *P* < 0.001; Fig. 1b).

### Prognostic model for DMFS

Patients were divided into four subgroups according to GTVnd and serum EBV DNA copy number: Group 1 (GTVnd ≤ 18.9 mL and EBV DNA ≤ 4000 copies/mL), Group 2 (GTVnd ≤ 18.9 mL and EBV DNA > 4000 copies/mL), Group 3 (GTVnd > 18.9 mL and EBV DNA ≤ 4000 copies/mL), and Group 4 (GTVnd > 18.9 mL and EBV DNA > 4000 copies/mL). In total, 415 (52.7%), 223 (28.3%), 62 (7.9%), and 87 (11.1%) patients belonged to Group 1, Group 2, Group 3, and Group 4, respectively, with corresponding 5-year DMFS rates of 96.0, 87.4, 96.6, and 73.8%, respectively. There was no significant difference in DMFS rates between Group 1 and Group 3 (*P* = 0.778). However, Group 4 had the lowest DMFS rate among the four groups (*P* < 0.05 for all); DMFS rate was significantly lower in Group 2 than in Group 1 and Group 3 (both *P* < 0.05).

Consequently, an integrated prognostic model was derived as follows: low risk (EBV DNA ≤ 4000 copies/mL regardless of GTVnd), moderate risk (GTVnd ≤ 18.9 mL and EBV DNA > 4000 copies/mL), and high risk (GTVnd > 18.9 mL and EBV DNA > 4000 copies/mL). Overall, 477 (60.6%), 223 (28.3%), and 87 (11.1%) patients were allocated to low-, moderate-, and high-risk groups, respectively, with the corresponding 5-year DMFS rates of 96.1, 87.4, and 73.8%, respectively. The DMFS rate of high-risk patients was significantly lower than those of patients in other risk groups (*P* < 0.001; Fig. 1c).

### The results of univariate and multivariate analyses

Table 2 shows the results of univariate and multivariate analyses for DMFS prediction. In univariate analysis, T category, GTVnd, serum EBV DNA copy number, and the prognostic model were significantly associated with DMFS (all *P* < 0.05). In multivariate analyses, GTVnd, serum EBV DNA copy number, and the prognostic model remained as independent prognostic factors for DMFS (all *P* < 0.05), but T category was no longer significant (*P* = 0.334).

**Table 2:** Univariate and multivariate analyses of prognostic factors for distant metastasis-free survival of patients with N1 nasopharyngeal carcinoma

Variable	Univariate analysis		Multivariate analysis	
	HR (95% CI)	*P* value	HR (95% CI)	*P* value^a^
Age	1.29 (0.79–2.10)	0.314	1.17 (0.67–1.89)	0.571
Gender	0.64 (0.35–1.17)	0.148	0.75 (0.61–1.45)	0.795
T category	2.01 (1.05–3.84)	0.035	2.42 (0.69–2.92)	0.334
GTVnd	3.02 (1.84–4.97)	< 0.001	2.22 (1.29–3.82)	0.004
EBV DNA copy number	4.61 (2.60–8.15)	< 0.001	3.24 (1.97–5.61)	< 0.001
Treatment method	3.05 (0.73–12.81)	0.128	2.12 (0.67–7.87)	0.697
Prognostic model	5.23 (3.14–8.69)	< 0.001	4.17 (2.34–7.59)	< 0.001

*HR* hazard ratio, *CI* confidence interval, *GTVnd* gross tumor volume of lymph nodes, *EBV* Epstein–Barr virus

^a^ *P* values are calculated using an adjusted Cox proportional hazards model

## Discussion

In the present study, we developed an integrated prognostic model that incorporates GTVnd and EBV DNA copy number to predict distant metastasis in NPC patients. Using this prognostic model, all patients were divided into three valid risk groups (*P* < 0.001): low-risk (5-year DMFS rate: 96.1%), moderate-risk (87.4%), and high-risk (73.8%). In multivariate analyses, the prognostic model was confirmed to be useful in predicting DMFS for patients with N1 NPC.

In general, patients with advanced N-category NPC are more likely to have a large GTVnd. However, patients with the same N-category disease also had different GTVnd and a vastly different prognosis. As demonstrated in the present study, patients with N1 NPC and GTVnd > 18.9 mL had significantly lower DMFS rate in comparison with those who had GTVnd ≤ 18.9 mL. Consistent with our present study, Lu et al. [11] prospectively analyzed 180 NPC patients and found that GTVnd was a significant prognostic factor for DMFS. These findings highlight the limitations of the current N category, which is mainly based on the largest nodal dimension and does not sufficiently reflect the tumor bulk in N1 disease. Furthermore, the addition of GTVnd may improve the accuracy in predicting distant metastasis for patients with N1 NPC.

Plasma EBV DNA copy number is a well-recognized biomarker for NPC [12, 13, 24]. Being consistent with previous studies, we confirmed that patients with high EBV DNA copy number (≥ 4000 copies/mL) had a > threefold increased risk of distant metastasis compared with patients with low EBV DNA copy number (< 4000 copies/mL). Furthermore, our results showed that patients with high EBV DNA copy number more commonly had large GTVnd and more frequently presented with advanced T category. This may suggest that EBV DNA load associates with tumor load in NPC patients [12, 17, 25]. In addition, although EBV DNA copy number and GTVnd as significant prognostic factors were both associated with DMFS, the HR of EBV DNA copy number was higher than that of GTVnd (3.24 vs. 2.22). This suggests that EBV DNA copy number may be a predictor of DMFS for patients with NPC.

Recently, several studies were conducted to stratify NPC patients into different groups based on the risk of distant metastasis to improve prognosis prediction [26–30]. For example, the study reported by Chen et al. [28] included age, N category, hemoglobin level, and lactate dehydrogenase level to the prognostic model, and found that the prognostic model was useful for predicting the risk of distant metastasis in patients with locally advanced NPC. Another study by Zhang et al. [30] incorporated fluor-18-fluorodeoxyglucose (^18^F-FDG) uptake value; N category was also confirmed to be a predictor for distant metastasis in their study. However, serum EBV DNA copy number, one of the most relevant factors in the prognosis of NPC [24], was excluded in these previous prognostic models. Furthermore, although the patients with N1 disease accounted for more than half of NPC patients, there was no related model to predict distant metastasis in this group of patients. In the present study, we extended this system by establishing a prognostic model that integrates GTVnd and EBV DNA copy number to predict DMFS in patients with N1 NPC. The prognostic model generated a balanced distribution, provided superior hazard discrimination compared with the GTVnd and EBV DNA copy number alone, and was confirmed to have significant prognostic value for DMFS.

Several studies have reported that neoadjuvant chemotherapy (NACT) could effectively reduce distant metastasis, but failed to observe any significant improvement in DMFS by NACT [31–33]. Two factors may explain these discrepancies. First, since the 5-year DMFS rate was up to 90% for patients with N1 NPC in the present study, the impact of NACT may be limited by the excellent distant control. Second, NACT was more commonly used for patients with high risk of distant metastasis, such as those with T3–4 NPC, a large tumor volume, and high EBV DNA copy number. In addition, the TNM staging system is currently the most reliable method for predicting treatment outcomes [7]. However, no significant influence of T category on distant metastasis was observed in the present study. This result may be due to the fact that patients with T3–4 NPC were more likely to receive NACT plus CCRT, which was reported to be able to prolong DMFS [31–33]. Therefore, it was not surprising that we failed to confirm the significance of T category in predicting DMFS in NPC patients.

Although our findings provides new insight on the prognostic model that incorporates GTVnd and EBV DNA copy number, several limitations should be noted. First, we did not include data of GTVp, which reflects tumor burden and has been demonstrated to strongly predict survival of NPC patients [34]. However, we performed stratified analysis based on EBV DNA copy number, which has been widely accepted as a significant biomarker of tumor burden [12, 35]. Another limitation is that we did not include the volume of the RLN in the GTVnd, as clear distinction between the RLN and primary tumor in NPC remains difficult [36, 37]. However, we did perform stratified analysis according to the volume of cervical lymph nodes and demonstrated that this volume was still a significant factor for DMFS.

## Conclusions

GTVnd and EBV DNA copy number are independent prognostic factors for predicting distant metastasis in NPC patients with N1 disease. Our prognostic model that incorporates GTVnd and EBV DNA copy number may be useful for predicting distant metastasis in this group of patients.
